# Self-Assembled Electret for Vibration-Based Power Generator

**DOI:** 10.1038/s41598-020-63484-9

**Published:** 2020-04-20

**Authors:** Yuya Tanaka, Noritaka Matsuura, Hisao Ishii

**Affiliations:** 1 Center for Frontier Science, Chiba University, Chiba, 263-8522 Japan; 2 Graduate School of Science and Engineering, Chiba University, Chiba, 263-8522 Japan; 3 Japan Science and Technology Agency, PRESTO, Saitama, 332-0012 Japan; 4 Molecular Chirality Research Center, Chiba University, Chiba, 263-8522 Japan

**Keywords:** Devices for energy harvesting, Electrical and electronic engineering, Electronic devices

## Abstract

The vibration-based electret generators (EGs) for energy harvesting have been extensively studied because they can obtain electrical energy from ambient vibrations. EGs exhibit a sandwich structure of electrodes surrounding an air gap and an electret, which is a dielectric material with a quasi-permanent electrical charge or dipole polarisation. Various charging processes have been developed because the surface charge density (σ) of the electret determines the output power of the device. However, such processes are considered to constitute a key productivity-limiting factor from the mass production viewpoint, making their simplification or elimination a highly desired objective. Herein, a model EG that does not require any charging process by utilising the spontaneous orientation polarisation of 1,3,5-tris(1-phenyl-1H-benzimidazole-2-yl)benzene (TPBi) is demonstrated. The surface potential (*V*_sp_) of an evaporated TPBi film has reached 30.2 V at a film thickness of 500 nm without using a charging process. The estimated σ of 1.7 mC m^−2^ is comparable with that obtained using a conventional polymer-based electret after charging. Furthermore, *V*_sp_ is considerably stable in environmental conditions; thus, TPBi can be considered to be “self-assembled” electret (SAE). Application of SAE leads to developing an EG without requiring the charging process.

## Introduction

Sensor nodes have become indispensable for obtaining an extensive spectrum of information related to factors such as personal health, human and animal locations and the condition of the natural and built environments, including buildings, bridges and tunnels. However, the problem associated with powering the wireless sensor networks has to be solved for maintaining a society permeated by such devices. Recently, energy harvesting from ambient sources, such as heat, electrical waves, light and vibrations, has attracted considerable attention as a substitute for batteries, which exhibit various problems in terms of, for instance, the necessity for regular replacement and their contribution to toxic waste. Among the ambient-source devices, vibration harvesters are considered to be favourable owing to the presence of vibration in several environments, enabling a potentially extensive range of applications^1^.

**Figure 1:**
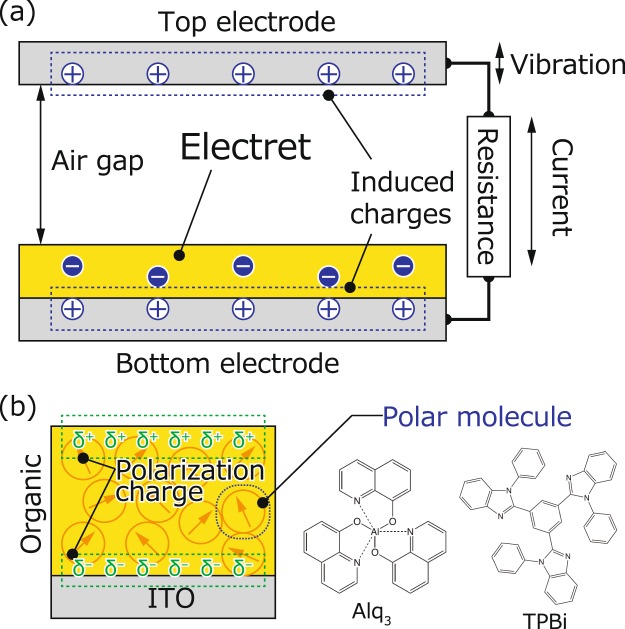
(**a**) Schematic of the vibration-based EG for energy harvesting. (**b**) Schematics of the Kelvin probe (KP) measurement of the SAE film and the Alq_3_ and TPBi chemical structures.

The vibration-based electret generators (EGs) are of particular interest for energy harvesting because they can provide relatively high output voltages even at low vibration frequencies ranging from few to several tens of hertz without the usage of any external bias source^1–4^. EGs typically exhibit a capacitor structure in which an electret and air gap are sandwiched between the top and bottom electrodes, as depicted in Fig. 1(a). An electret is a dielectric material with a quasi-permanent electrical charge or dipole polarisation^5^. These are indispensable in EGs because charges are induced by the electric field of the electret, eliminating the external bias for charging the capacitor^3,6,7^. An AC current is generated by the variance in air gap due to the out-of-plane (OP) vibration (Fig. 1(a)) when the amount of induced charge changes. Because the output power (*P*) is proportional to the square of the surface charge density of the electret (σ)^2,8^, enhancing the σ of the electret is vital for improving the EG device performance (see Note A, Supplementary Information).

Furthermore, the electret materials can be classified into polymer-based and inorganic-based electrets^2,5^. In general, inorganic electrets exhibit higher σ when compared with that exhibited by polymer electrets with shorter retention times^9^. The interest in polymer electrets has increased because of their low fabrication temperatures and high compatibilities with various substrate materials^3,8,10–12^. However, regardless of the electret type, a charging process is definitely required to fabricate the electret using its constituent dielectric materials. In corona charging, which is one of the most extensively used electret forming techniques, charges are transferred from the corona ions to the dielectric surface by attracting ions using the electrostatic field between a grid and a substrate^2,5^. Kashiwagi *et al*. reported that a high σ of 2.0 mC m^−2^ was produced in an aminosilane-doped cyclic transparent optical polymer (CYTOP) film via corona charging^10^. Although corona charging is useful for producing high σ electrets, it is necessary to apply a high voltage to a needle and a grid for forming corona ions. It is also difficult to uniformly charge the dielectric film and optimise various parameters, such as the applied voltages and distances, among the needle, grid and sample. Alternative fabrication techniques, including electron beam, photoexcitation, X-ray or thermal excitation and ion implementation, that achieve a high σ value without corona charging have been proposed^2,5,13–18^. However, all these charging processes involve factors that limit the productivity in terms of mass production; thus, simplification or elimination of the charging process would be exceptionally useful.

In fact, spontaneous polarisation has been reported in thin films for some types of molecules. For example, small polar molecules impinging on a cold surface from the gas phase have been observed to be capable of sticking onto a substrate, forming a charged film without a charging process^19–23^. After almost 50 years of research^24,25^, this vital phenomenon has been considered to originate from a partial orientation of the permanent dipoles of the molecules. In general, the potential of the resulting film is considered to be linearly proportional to its thickness and is observed to be stable with time over a period of hours^23^. Surprisingly, in case of 1-butanol, the potential of the film easily exceeds 100 V^19^. These characteristics strongly imply that this phenomenon can be applied to the fabrication of the charged films without using a charging process. However, applying such films to actual devices is difficult because low temperatures, particularly several tens of Kelvins, are necessary to absorb small molecules.

In 2002, Ito *et al*. applied the Kelvin probe (KP) technique to a vacuum-evaporated film of tris-(8-hydroxyquinolinato) aluminium (Alq_3_) (Fig. 1(b)), which is one of the most famous light-emitting and electron transport materials in organic light-emitting diodes (OLEDs). They reported that the surface potential (*V*_sp_) of the film increased linearly with the film thickness, eventually reaching 28 V at a thickness of 560 nm and room temperature^26^. The second harmonic generation (SHG) measurement revealed that this giant surface potential (GSP) can be attributed to the spontaneous orientation of the permanent dipole moment of the Alq_3_ molecule^26,27^. These results were directly consistent with the inducement of a surface charge density of σ = ±1.4 mC m^−2^ on both the film surface and the reverse side without using a charging process in the manner denoted in Fig. 1(b).

This notable phenomenon can be observed using various polar molecules, particularly in OLEDs^28–30^. In case of tris(7-propyl-8-hydroxyquinolinato) aluminium (Al(7-Prq)_3_), σ reaches 3.1 mC m^−2^, determined using the displacement current measurement, which is a capacitance–voltage measurement technique^31,32^. Further, the decay rate of the evaporated Alq_3_ film was estimated to be approximately 10 years for 10% loss in dark and vacuum conditions^33^. These results indicate that such GSP-demonstrating molecules can be considered to be “self-assembled electrets (SAEs)” because they are spontaneously ordered and form charged films without requiring a charging process. This elimination of the charging process through the application of SAE materials would significantly aid EG fabrication.

However, one problem associated with the usage of SAEs while manufacturing EGs is the instability of *V*_sp_ with respect to light irradiation. In case of Alq_3_, *V*_sp_ can be completely eliminated under illumination when the photon energy exceeds the absorption edge of the material (2.8 eV)^26,33–35^. This occurs because excitons are created by light absorption and are dissociated owing to the internal field in a film, resulting in the compensation of polarisation charge by the photo-generated holes and electrons. However, Tanaka *et al*. reported that GSP decay could not be observed in bathocuproine (BCP)^36^, suggesting the inefficient generation of photocarriers in BCP films relative to Alq_3_ owing to the wider optical bandgap of the former (3.5 eV)^37^. This indicates that an SAE with a wide optical bandgap can be used to overcome the problem of light stability.

In this study, we developed a novel modelled EG that does not require a charging process using the SAE 1,3,5-tris(1-phenyl-1H-benzimidazol-2-yl)benzene (TPBi) (Fig. 1(b)). The σ of TPBi is higher than that of BCP, and TPBi is considerably more stable than BCP under atmospheric conditions^29,38,39^. Because the optical bandgap of TPBi (3.5 eV) is wider than that of Alq_3_^40^, we expect TPBi to exhibit a considerably low light absorption coefficient in visible light and, correspondingly, a large light stability. Further, we observed *V*_sp_ values that exceeded 30 V at 500 nm in an evaporated TPBi film without the requirement of a charging process. The estimated σ was 1.7 mC m^−2^, comparable to that of CYTOP following corona charging (2.0 mC m^−2^)^10^. As expected, *V*_sp_ was stable under both dark and light-illuminated conditions. Based on these advantages, we fabricated a TPBi SAE-based modelled EG without using a charging process that resulted in a root mean square (rms) current of a few nanoamperes based on electrode vibration.

For *V*_sp_ measurement, the KP measurement system (UHVKP020, KP Technology) has been used in this study. This system includes an external voltage (*V*_ext_) source and measures the AC current produced when a probe close to the sample surface vibrates in a sinusoidal manner. Because this probe can be regarded as the vibration electrode of OP vibration EG, the output current of the model EG was measured by oscilloscope through an I/V amplifier after the external voltage source was eliminated from the KP measurement system.

## Results and Discussion

### Surface potential measurements

**Figure 2:**
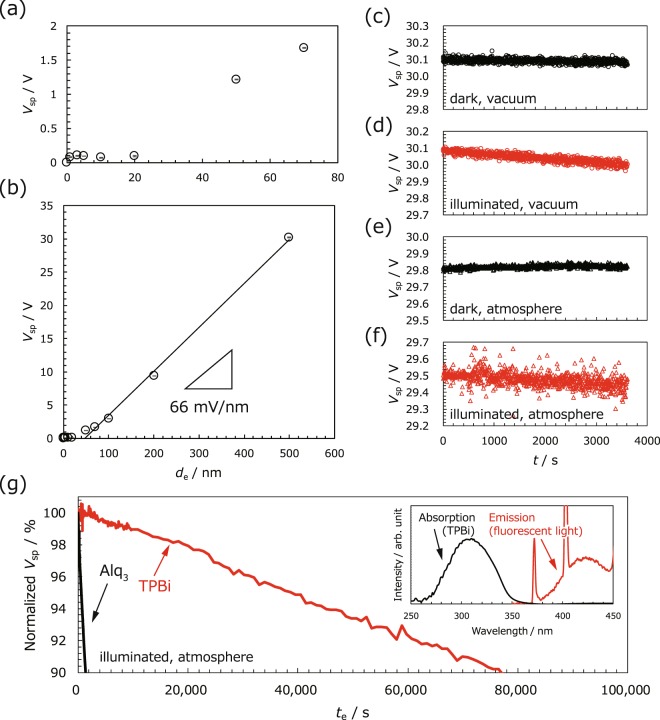
Variation of *V*_sp_ of the TPBi film in (**a**) thin and (**b**) thick regions. *V*_sp_ is plotted against the surface potential of ITO. (**c–f**) Variation of *V*_sp_ in dark vacuum, illuminated vacuum, dark atmosphere and illuminated atmosphere conditions, respectively. (**g**) Comparison of the *V*_sp_ stability between Alq_3_ and TPBi in an illuminated atmosphere. Inset indicates the absorption spectrum of TPBi and the emission spectrum of the irradiated light.

We initially investigated the evolution of the surface potential of the TPBi film. Figure 2(a,b) denote the variation in *V*_sp_ as a function of the film thickness (*d*_e_) in thin (0–80 nm) and thick regions (0–600 nm), respectively (in the figures, an upward shift can be defined as a positive change in the surface potential). Figure 2(a) depicts that increasing the thickness to 1 nm shifts *V*_sp_ upward by 77.0 ± 4.3 mV because of interface dipole formation^41^. This shift is followed by an almost constant plateau in *V*_sp_ for up to 20 nm, which often appears in organic films denoting GSP^26^. The constant *V*_sp_ in this thickness region suggests random orientation of TPBi. Then the *V*_sp_ jumps to 1.2 V at the film thickness of 50 nm, indicating that the permanent dipole of TPBi starts to be oriented perpendicular to the substrate. In other words, not all the adjacent molecules form antiparallel orientation of their dipole moment in plane. This can be evidenced by variation of *V*_sp_ in thick region; It increases linearly over a wide range of thicknesses (50–500 nm) and finally reaches 30.20 ± 0.03 V at a thickness of 500 nm without the requirement of any charging process. This result clearly means the occurrence of spontaneous orientation polarisation in the TPBi film^29^. The solid line in Fig. 2(b) denotes the fitted curve in the thickness region of 50–500 nm based on which an electric field in a film (*E*_e_) and σ can be estimated to be 66 mV nm^−1^ and 1.7 mC m^−2^, respectively (Eq. (S19), Supplementary Information). Notably, a high σ value for the TPBi film, comparable to that obtained in CYTOP (2.0 mC m^−2^), was achieved using simple vacuum evaporation in the dark instead of a charging process. Note here that *V*_sp_ of TPBi (30.2 V) is much smaller than that of the polymer-based electret (typically, 1 kV)^10^. This is because the film thickness of the former (500 nm) is much less than that of the latter (15 μm) in this experiment. In other words, we can expect that *V*_sp_ of TPBi reaches approximately 900 V at 15 μm because it is proportional to the film thickness (see Eq. (S7), Supplementary Information), suggesting that TPBi is suitable for practical applications.

There are two possible origins of the charge resulting in the buildup of large *V*_sp_. First, it seems reasonable to suppose that the charges are distributed at the vicinity of the TPBi surface due to trapping. Second reason is the partial alignment of the permanent dipole of TPBi. Since TPBi was deposited without any charging process in dark conditions, in the former case, the charge may originate from thermal electron generated by heating for TPBi evaporation and/or thermally excitation across the HOMO/LUMO gap (HOMO and LUMO indicate the highest occupied and lowest unoccupied molecular orbitals, respectively). Thus, the former case is not likely to occur because the deposition temperature of TPBi at vacuum evaporation is not high and the HOMO/LUMO gap of TPBi (ca. 3.5 eV) is much larger than the thermal energy at room temperature (ca. 26 meV). Even if the charges are generated by heating and/or thermal excitation, the electric field formed in TPBi is not in the direction to move the charges to the surface, but to drive them into the bulk, leading to the decrease of *V*_sp_. In contrast, as discussed in the introduction, SHG measurements reveal that GSP appeared in Alq_3_ film originates from spontaneous orientation of dipole moment of the molecule^26,27^, and previous reports demonstrate that GSP is not formed in non-polar molecules^26,28,29^. Thus, we conclude that the charge forming large *V*_sp_ in TPBi film originates from spontaneous orientation polarisation similarly to the Alq_3_ case.

From the obtained σ, the average order parameter <cos ϴ> of the TPBi molecule can be calculated, where ϴ denotes the tilting angle of the dipole moment with respect to the surface normal. By assuming a permanent dipole moment of 7.0 D and volume per molecule of 0.96 nm^3^ ^42^, <cos ϴ> is estimated to be approximately 0.07, indicating that the average orientation of TPBi is almost random and slightly ordered. This indicates that there is sufficient room for enhancing the value of σ up to 24.3 mC m^−2^ if the TPBi molecules can be perfectly aligned.

Molecular orientation has become the focus of attention in the field of organic semi-conductor research because it strongly affects the performance in terms of the mobility and efficiency of devices such as OLEDs^43–45^, organic solar cells^46,47^, and organic field-effect transistors^48,49^. Furthermore, the driving forces of molecular orientation remain uncertain in terms of solid-state physics, leading to several researches focusing on OLED materials^44,45,50–53^. Additionally, various experiments and simulations have been conducted over the previous decade with an objective of understanding the molecular orientation of SAE materials, which are also known to form amorphous films^28,29,32,42,54,55^.

In case of Alq_3_, Noguchi *et al*. demonstrated that a molecular head-to-tail configuration requires a lower potential energy on an average when compared with that required by an antiparallel configuration and that both the energies are comparable with the thermal energy, indicating that the partial orientation can be attributed to the dipole–dipole interaction of polar molecules^29^. Isoshima *et al*. suggested that the differences in orientation between Alq_3_ and Al(7-Prq)_3_ can be attributed to their respective molecular shapes^32^. In 2016, Jäger *et al*. experimentally demonstrated that the degree of orientation polarisation of Alq_3_ increased as the Alq_3_ concentration decreased in 4,4-bis[N-(1-naphthyl)-N-phenylamino]-bipenyl (NPB)^54^. They attributed this improved orientation to the attenuation of the dipole–dipole interaction with increasing intermolecular distance between the Alq_3_ molecules. Their model was further supported by Friederich *et al*.^42^, who simulated the physical vapour deposition process of the SAE materials and observed that the electrostatic interaction between the dipole moments limited the GSP strength. Further, they identified the short-range van der Waals interactions between molecules and the surface during deposition as the driving force behind the anisotropic orientation. In this way, the importance of dispersive forces has been recently noted^52,53,55^ even though a consensus model for explaining the spontaneous orientation of molecules has not yet been constructed. In addition to the models mentioned above, intriguing clue may be yielded from the findings in strongly correlated electron system^56–59^. In any case, further investigation to clarify the origin of spontaneous orientation will be useful to develop methods for controlling the molecular orientation and the synthesis of new molecules having high σ.

In addition to our evaluations of the pristine film, the stability of *V*_sp_ can be measured in various conditions. Figure 2(c–f) denote the results of measurements that have been performed in dark vacuum, illuminated vacuum, dark atmosphere and illuminated atmosphere conditions, respectively. We can observe that there is almost no variation in *V*_sp_ (<0.01 V) over the measurement range under both the dark conditions (Fig. 2(c,e)). In contrast, *V*_sp_ slightly decreases by approximately 0.1 V (0.3% decrease over a period of 1 h) under both the illumination conditions (Fig. 2(d,f)).

To confirm our expectations, the *V*_sp_ stability of TPBi was directly compared with that of Alq_3_ in illuminated atmosphere, as depicted in Fig. 2(g). The vertical and horizontal axes denote the *V*_sp_ normalised by the initial values and exposure time (*t*_e_), respectively. The retention time of 10% loss in Alq_3_ was 1.4 × 10^3^ s. In contrast, the retention time of TPBi was 7.6 × 10^4^ s, 54 times longer than that in Alq_3_. These results confirm our expectations and indicate that the *V*_sp_ stability of TPBi originates from the wide optical bandgap, resulting in weak visible light absorption^34,36,40^. The moderate decrease observed with respect to *V*_sp_ in TPBi under illumination can be explained by the light absorption at the peak wavelength (372 nm) of the fluorescent lamp in our laboratory, which is a wavelength that agrees with one of the absorption edges of TPBi (see the inset in Fig. 2(g)). The electrons and holes are slowly provided even in TPBi, resulting in compensation of polarization charge of the film.

### Characteristics of the generated current

**Figure 3:**
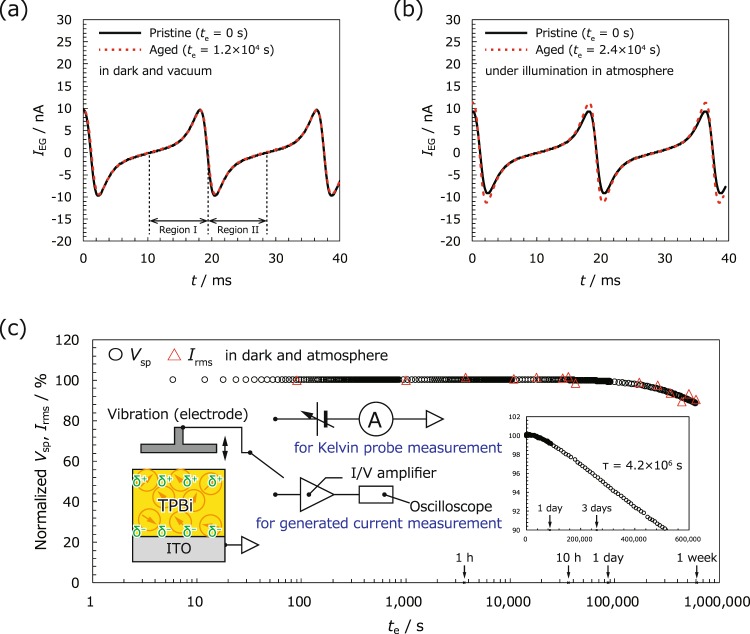
Time dependence of the generated current owing to electrode vibration (**a**) in dark vacuum at *t*_e_ = 0 s (solid line) and 1.2 × 10^4^ s (dotted line) and (**b**) under illumination in atmosphere at *t*_e_ = 0 s (solid line) and 2.4 × 10^4^ s (dotted line). (**c**) Variation in normalised *V*_sp_ (circles) and *I*_rms_ (triangles) as a function of *t*_e_ (semi-log plot). The insets indicate the *t*_e_ dependence of normalised *V*_sp_ and *I*_rms_ as a linear plot and the setups used to obtain the *V*_sp_ and *I*_rms_ measurements.

Further, we evaluate the current generation characteristics of the modelled TPBi-based EG devices, obtained by measuring the generated current using the KP system under *V*_ext_ = 0 V. The probe was vibrated using *d*_0_ = 216 µm, *∆d* = 82 µm and *f* = 55 Hz, corresponding to a maximum acceleration of 9.79 m s^−2^. The solid line in Fig. 3(a) denotes the variation in output current (*I*_EG_) as a function of time under the dark vacuum condition. An rms AC current (*I*_rms_) of 4.7 nA can be clearly observed with a frequency identical to that of the probe (1/*f* = 18.2 ms). This result demonstrates that a modelled EG with a TPBi SAE will generate a current because of electrode vibration without requiring a charging process.

The *I*_EG_ curve can be divided into two regions, I (10.5–19.3 ms) and II (19.3–28.7 ms), with the times at which *I*_EG_ = 0 nA being set as the boundaries. Subsequently, the shape of the *I*_EG_ curve can be explained as follows. In region I, *I*_EG_ gradually increases over *t* from 0 nA because of the probe approach. Further, *I*_EG_ drastically increases and reaches its maximum, as reflected by the rapid change in d*C*_KP_/d*t*, where *C*_KP_ indicates the capacitance in model EG (see Eq. (S14) and Supplementary Fig. S2(a), Supplementary Information). Finally, *I*_EG_ suddenly decreases to 0 nA as the probe reduces its speed and stops momentarily at its closest approach to the sample. Similar changes can be observed when the probe leaves the sample, producing a point-symmetrical curve with respect to *t* = 19.3 ms in region II (see Supplementary Fig. S2(b), Supplementary Information). In this way, the waveform of the generated current was explained based on the basics of KP and equivalent circuit model for vibration-based power generator (see Notes A and B, Supplementary Information)^60,61^. However, further analysis, including contribution of parasitic capacitance, is required to fully reproduce the experimental data^62^.

The dotted line in Fig. 3(a) denotes the current generated after the device was kept in dark vacuum for 1.2 × 10^4^ s (ca. 3.3 h). The solid and dotted lines in Fig. 3(b) show *I*_EG_ just after the device was exposed to air, i.e., at *t*_e_ = 0 and 2.4 × 10^4^ s (ca. 6.7 h), respectively. The *I*_EG_ curves coincide under all the measurement conditions, suggesting that a favourable EG stability can be achieved using TPBi as an SAE.

Finally, to accurately evaluate the long-term stabilities of *V*_sp_ and *I*_rms_, a model EG with a TPBi thickness of 200 nm was prepared following an identical fabrication process as that mentioned above. Figure 3(c) depicts the variation in *V*_sp_ and *I*_rms_ as functions of *t*_e_ in a dark atmosphere. Herein, *V*_sp_ and *I*_rms_ are plotted after normalisation using their pristine values. The insets denote a linear plot of Fig. 3(c) and the measurement setups used for performing the KP and generated current measurements.

*V*_sp_ and *I*_rms_ change synchronously and are considerably stable (<1% loss) at 1.0 × 10^5^ s (>24 h). Subsequently, both of them gradually decrease to 95% at *t* = 2.9 × 10^5^ s (ca. 81 h), as depicted in the inset of Fig. 3(c). The retention time of the 10% loss of *V*_sp_ is approximately 5.2 × 10^5^ s (ca. 144 h). Further, the decrease in *V*_sp_ and *I*_rms_ can be explained by the adsorption of counter ions and water molecules from air^26,33^. Although the measurement time was not sufficiently long to discuss the functional type of the decay process in Fig. 3(c), an exponential decay is likely to occur in *V*_sp_ because negative atmospheric ions are likely to stick onto the positive surface of the TPBi film, blocking subsequent ions from accumulating at the same position. By fitting the *V*_sp_ decay to an exponential function, an estimated time constant (*τ*) of 4.2 × 10^6^ s (ca. 49 days) can be obtained. These results indicate that a model EG with a TPBi SAE will exhibit a relatively favourable lifetime even under standard atmospheric conditions.

In the Alq_3_ film, Sugi *et al*. showed that the decay rate of the *V*_sp_ in vacuum (10% decay in 10 years) is considerably longer than that in air (10% decay in a few hundreds of hours)^33^. It is expected that both *V*_sp_ and *I*_rms_ are almost stable in the case that the sample is kept in vacuum. Thus, the encapsulation techniques that have long been used to study the OLEDs and microelectromechanical systems should prove to be considerably useful for the SAE-based EGs^63,64^. However, further investigation will be required to clarify the detailed origin of *V*_sp_ degradation for facilitating the synthesis of SAE materials with high σ and long *τ* values that are compatible with the practical application of such devices.

## Conclusion

In summary, we have developed a novel modelled EG that does not require a charging process because of the application of spontaneous orientation polarisation of the TPBi molecule. The *V*_sp_ of TPBi was observed to exceed 30 V at 500 nm without a charging process, and σ of 1.7 mC m^−2^, comparable to that of the polymer-based electrets after corona charging, was obtained. Furthermore, *V*_sp_ was stable in both the vacuum and atmosphere conditions, particularly in the dark. We used these favourable characteristics of the TPBi molecule to develop a model EG without a charging process that could generate rms current of a few nanoamperes with a maximum acceleration of 9.79 m s^−2^ and a frequency of 55 Hz. By considering the order parameter and *V*_sp_ stability, we observed that there was sufficient room to improve the device performance of SAE-based EGs by a) manipulating the molecular orientation and b) encapsulating the device. We believe that the application of SAEs in the development of EGs without the requirement of charging processes will provide novel opportunities for supplying cheap and clean electrical energy to various distributed network devices that are becoming increasingly important to sustain the connectedness of our society.

### Experimental section

TPBi (sublimed, purity >99.5%) was purchased from Luminescence Technology Corp. The sample preparation was conducted in an evaporation chamber with a base pressure of 4 × 10^−4^ Pa. Further, the KP measurements were performed in a measurement chamber directly connected to the evaporation chamber in either vacuum or atmosphere. To perform the measurements under illumination conditions, the fluorescent (indoor) light was irradiated through one of the glass viewing ports of the measurement chamber. The spectrum of the light is shown in the inset of the Fig. 2(g). Furthermore, the TPBi film was incrementally formed on the ITO at a typical deposition rate of 2 Å/s, with the *V*_sp_ measurements being conducted *in situ* during each step of the deposition. Because GSP usually decays upon light absorption by the film^26^ all the aforementioned procedures, except for the measurement conducted under illumination, were conducted in the dark or under red light illumination. All experiments were carried out at room temperature. The thickness of the TPBi film was monitored using a quartz microbalance, and an ultra-high vacuum KP system (UHVKP020, KP Technology) was used for performing the *V*_sp_ measurements. To measure the current generated by the vibration of the KP probe, which was used as the vibrational electrode for the model EG device, an I/V amplifier (SR570, SRS) was connected to the probe; furthermore, its output voltage was detected using an oscilloscope (TDS 2001C, Tektronix). *V*_ext_ was set to zero while measuring the generated current.

## Supplementary information

Supplementary Information.

## Data Availability

All data generated or analysed during this study are included in this published article.
