# Supplementary material for: Self-Assembled Electret for Vibration-Based Power Generator
Source: Sci Rep. 2020 Apr 20;10:6648. doi: 10.1038/s41598-020-63484-9 (PMC7170925; doi:10.1038/s41598-020-63484-9)
Supplement: Supplementary file 1 — Supplementary Information. [file 41598_2020_63484_MOESM1_ESM.pdf]

Supplementary Information

Self-Assembled Electret for Vibration-Based Power Generator

Yuya Tanaka[1,2,3], *Noritaka Matsuura*[2], *and Hisao Ishii*[1,2,4]

[1]Center for Frontier Science, Chiba University, Chiba, 263-8522, Japan

[2]Graduate School of Science and Engineering, Chiba University, Chiba, 263-8522, Japan

[3]Japan Science and Technology Agency, PRESTO, Saitama, 332-0012, Japan

[4]Molecular Chirality Research Center, Chiba University, Chiba, 263-8522, Japan

Correspondence and requests for materials should be addressed to Y.T. (email: y-tanaka@chiba-u.jp)

Note A. Equivalent circuit model for electret generators (EGs)

In general, there are two types of EGs, which generate AC current due to out-of-plane (OP) vibration (see Figure 1(a)) and in-plane (IP) vibration, where the interdigital electrodes and patterned electret layers undergo IP motion with respect to each other. The output power (*P*) of the IP vibration EG is proportional to the square of the surface charge density of the electret (σ) as reported.[1,2] Similarly, a $P \propto \sigma^2$ relation is maintained in OP vibration EGs as follows.

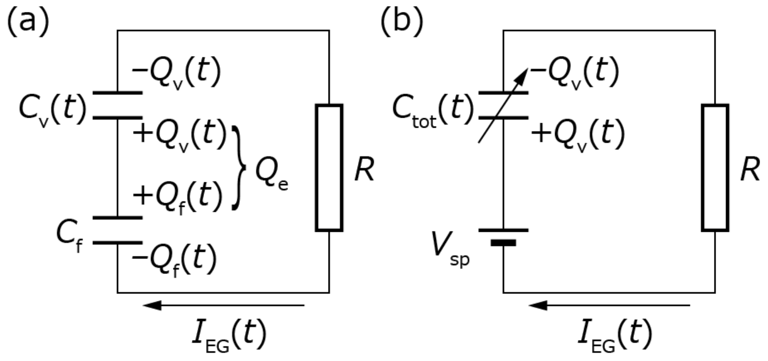

Supplementary Figure S1. Equivalent circuits of EG: (a) in a static condition and (b) during out-of-plane vibration.

An OP vibration-based EG comprises a variable and a fixed capacitor that are connected in series, as depicted in Supplementary Figure S1(a).[3,4] The variable and fixed capacitors comprise a top electrode/air gap/electret surface and an electret surface/electret layer/bottom electrode, respectively. In Supplementary Figure S1(a), *t* and *R* indicate the time and external load resistance, respectively. Further, the capacitances of the variable and fixed elements are given as $C_v(t)$ and C_f, respectively. The respective stored charges are $Q_v(t)$ and $Q_f(t)$; accordingly, the total amount of charge in the electret (Q_e) can be expressed as

$$Q_e = Q_v(t) + Q_f(t) \tag{S1}$$

$$= \sigma S, \tag{S2}$$

where S denotes the active area of the device. The circuit dynamics during EG vibration can be obtained using Kirchhoff's law as follows:

$$\frac{Q_{\mathrm{v}}(t)}{C_{\mathrm{v}}(t)} - \frac{Q_{\mathrm{f}}(t)}{C_{\mathrm{f}}} + RI_{\mathrm{EG}}(t) = 0, \tag{S3}$$

where $I_{\mathrm{EG}}(t)$ denotes the current generated owing to vibration. By substituting Q_{f} from Equation (S1) into Equation (S3), we can obtain the overall governing behaviours of an EG with OP vibration as follows:

$$I_{\mathrm{EG}}(t) = \frac{dQ_{\mathrm{v}}(t)}{dt} \tag{S4}$$

$$= \frac{1}{R}\left(V_{\mathrm{SP}} - \frac{Q_{\mathrm{v}}(t)}{C_{\mathrm{tot}}(t)}\right), \tag{S5}$$

where V_{sp} and $C_{\mathrm{tot}}(t)$ denote the voltage at the electret surface and the total capacitance of the device, respectively, which can be given as

$$V_{\mathrm{SP}} = \frac{Q_{\mathrm{e}}}{C_{\mathrm{f}}} \tag{S6}$$

$$= \frac{Q_{\mathrm{e}}}{\varepsilon_{\mathrm{e}}\varepsilon_0 \frac{S}{d_{\mathrm{e}}}}, \tag{S7}$$

$$C_{\mathrm{tot}}(t) = \frac{C_{\mathrm{f}}C_{\mathrm{v}}(t)}{C_{\mathrm{f}} + C_{\mathrm{v}}(t)}, \tag{S8}$$

where ε_{e}, ε_0, and d_{e} denote the relative permittivity of the electret, permittivity of vacuum, and thickness of the electret, respectively. Based on this observation, an equivalent circuit during EG vibration can be illustrated, as depicted in Supplementary Figure S1(b).[3] The generated power ($P(t)$) of the circuit can be calculated based on $P(t) = RI(t)^2$. For cases in which the distance between the top electrode and electret surface varied sinusoidally, $C_{\mathrm{v}}(t)$ can be obtained as

$$C_{\mathrm{v}}(t) = \frac{\varepsilon_0 S}{d_0 + \Delta d \sin(\omega t + \phi)}, \tag{9}$$

where d_0, Δd, ω and ϕ are the distance between the top electrode and the electret surface in

the absence of vibration, amplitude, angular frequency and phase of vibration, respectively. Here, ω is equal to $2\pi f$, where f denotes the vibration frequency. In this equivalent circuit model, no current is observed to flow in a static condition (no vibration). By assuming that a static condition is maintained at $t = 0$, $Q_v(0)$ can be given by

$$Q_{\mathrm{v}}(0) = \frac{C_{\mathrm{v}}(0)}{C_{\mathrm{f}} + C_{\mathrm{v}}(0)} Q_{\mathrm{e}}. \tag{S10}$$

Equations (S4) and (S5) can be analytically solved as

$$Q_{\mathrm{v}}(t) = \left\{ \frac{V_{\mathrm{sp}}}{R} \int_0^t e^{-t_{\mathrm{RC}}(t)} dt + Q_{\mathrm{v}}(0) \right\} e^{t_{\mathrm{RC}}(t)}, \tag{S11}$$

$$t_{\mathrm{RC}}(t) = -\int_0^t \frac{1}{RC_{\mathrm{tot}}(t)} dt, \tag{S12}$$

where $t_{\mathrm{RC}}(t)$ indicates time variation of the time constant in the equivalent circuit (Supplementary Figure S1(b)). By inserting the Equation (S2) and (S6) to (S11), a $Q_v(t) \propto \sigma$ relation is obtained; i.e., the P (= RI_{EG}^2) $\propto \sigma^2$ relation is also maintained in EGs exhibiting OP vibration.

Note B. Kelvin probe method

The KP method is a powerful tool for measuring the contact potential difference (V_{CPD}) between a sample and a reference electrode. The KP measurement system includes an external voltage (V_{ext}) source and an ammeter. The KP method measures the AC current ($I_{KP}(t)$) produced when a probe close to the sample surface vibrates in a sinusoidal manner. In a metal sample, a displacement current $I_{KP}(t)$ will flow through a condenser with a probe-to-metal capacitance ($C_{KP}(t)$) that can be given by

$$C_{\mathrm{KP}}(t) = \frac{\varepsilon_0 S}{d_{\mathrm{KP}}(t)} \tag{S13}$$

$$= \frac{\varepsilon_0 S}{d_0 + \Delta d \sin(\omega t + \phi)}, \tag{S14}$$

where $d_{KP}(t)$ denotes the distance between the probe and metal. Thus, $I_{KP}(t)$ is obtained by

$$I_{KP}(t) = (V_{CPD} - V_{ext})\frac{dC_{KP}(t)}{dt} \tag{S15}$$

$$= -\varepsilon_0 S(V_{CPD} - V_{ext})\frac{\omega\Delta d\cos(\omega t + \phi)}{\{d_0 + \Delta d\sin(\omega t + \phi)\}^2}. \tag{S16}$$

By assuming $\Delta d \ll d_0$, the equation for $I_{KP}(t)$ can be reduced to a simple sinusoidal function that can be given as follows:

$$I_{KP}(t) = -\varepsilon_0 S(V_{CPD} - V_{ext})\frac{\omega\Delta d\cos(\omega t + \phi)}{{d_0}^2}. \tag{S17}$$

Subsequently, V_{CPD} can be obtained from V_{ext} when $I_{KP}(t)$ = 0 A.

For an SAE film on indium–tin–oxide (ITO), V_{sp} can be estimated by subtracting the ITO contribution (−316.0 ± 6.8 mV) from the measured V_{CPD} based on which σ can be calculated as

$$\sigma = \frac{\varepsilon_e\varepsilon_0 V_{SP}}{d_e} \tag{S18}$$

$$= \varepsilon_e\varepsilon_0 E_e, \tag{S19}$$

where E_e denotes the electric field in a film, which can be easily estimated based on the slope of the dependence of V_{sp} on the film thickness.

Notably, both the currents generated in EGs and those flowing during the KP measurement are the AC currents originating from the variation of the capacitors, as presented using Equations (S9) and (S14), respectively. Therefore, the generated current due to vibration can be evaluated by utilising the probe in the KP as a vibration electrode with V_{ext} = 0 V.

Note C. Waveform of the generated current in a model EG

As mentioned above, the output current of the model EG can be measured using the KP system under V_{ext} = 0 V because the probe works as the vibration electrode of (model) EG.

Thus, the shape of $I_{EG}(t)$ can be simulated using Equation (S16) assuming $V_{ext} = 0$ V. The parameters presented in (Table S1) have been used in the simulation, and S, d_0, Δd and f were the actual set values for the KP system. d_0 and Δd were the obtained values in our KP system by measuring the capacitance under probe vibration. The ε_e of TPBi (2.87) was used to perform the simulation.[5]

Table S1. Parameters used in the electrical model.

Parameter	Unit	Value
ε_e	—	2.87
d_e	nm	500
S	mm^2	28.3
d_0	µm	216
Δd	µm	82
f	Hz	55

Supplementary Figure S2(a) depicts the simulated C_{KP} (Equation (S14)) obtained by varying $\Delta d/d_0$ from 0.09 to 0.94. In this simulation, the probe approached the sample from $0 \leq t < 9.1$ ms and was eliminated from 9.1 ms $\leq t <$ 18.2 ms. For ensuring simplicity, C_{KP} is normalised by the maximum value in Supplementary Figure S2(a). At $\Delta d/d_0 = 0.09$, C_{KP} varies sinusoidally with time, reaching the maximum and minimum values when the distance between the probe and sample is the shortest (t = 9.1 ms) and longest (t = 0 ms), respectively. When $\Delta d/d_0$ increases beyond 0.09, a peak structure begins to appear in the C_{KP} curve because the contribution of the $\Delta d \sin(\omega t + \phi)$ term in Equation (14) increases. When $\Delta d/d_0 \approx 1$, the relation $C_{KP} \propto 1/(1 + \sin \omega t)$ is approximated. Therefore, C_{KP} is stable and close to zero, except in the region where a peak can be clearly observed.

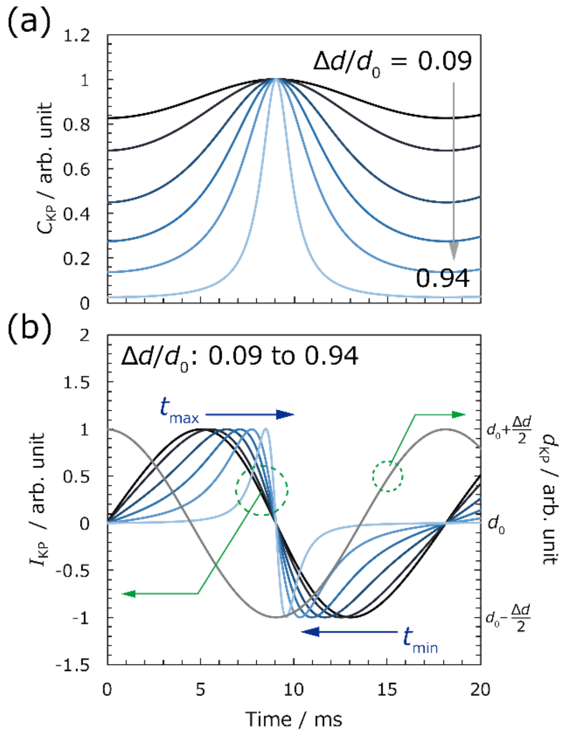

Supplementary Figure S2. The simulated results of (a) normalised $C_{KP}(t)$ and (b) normalised $I_{KP}(t)$ results as a function of $\Delta d/d_0$ ratios of 0.09, 0.19, 0.38, 0.57, 0.76 and 0.94. t_{max} and t_{min} indicate the time at which I_{KP} is maximised and minimised, respectively. $d_{KP}(t)$ is also plotted in Supplementary Figure S2(b).

Supplementary Figure S2(b) depicts the simulated values of I_{KP} (Equation (S16)) at various values of $\Delta d/d_0$ (left ordinate) and d_{KP} (right ordinate) as a function of time. The maximum ($d_0 + \Delta d/2$) and minimum ($d_0 - \Delta d/2$) values of d_{KP} indicate the point at which the distance between the probe and sample is the largest (d_{KP}(max)) and smallest (d_{KP}(min)), respectively. When $\Delta d/d_0 = 0.09$, i.e., the point at which $\Delta d \ll d_0$ is almost maintained constant, I_{KP} changes sinusoidally, as predicted in Equation (S17). During each period, $I_{KP} = 0$ can be observed when $t = 0$ and 9.1 ms because the probe nearly stops at d_{KP}(max) and d_{KP}(min), causing the $\mathrm{d}C_{KP}(t)/\mathrm{d}t$ term in Equation (S15) to vanish (see also Supplementary Figure S2(a)). Because of the relations $I_{KP} \propto \cos \omega t$ and $d_{KP} \propto \sin \omega t$ obtained from Equations

(S17) and (S14), respectively, I_{KP} reaches the maximum (I_{KP}(max)) and minimum (I_{KP}(min)) values when d_{KP} crosses d_0 with decreasing (t_{max}) and increasing (t_{min}), respectively.

As $\Delta d/d_0$ increases, t_{max} and t_{min} increases and decreases, respectively, and the shape of the I_{KP} curve becomes asymmetric with respect to both because the capacitance is no longer sinusoidal with respect to time in this condition, as depicted in Supplementary Figure S2(a); conversely, $\Delta d/d_0$ is almost stable at relatively low values when the probe is located far from the sample and drastically increases and decreases when the probe is located in the vicinity of the sample. Therefore, I_{KP}(max) and I_{KP}(min) can be observed close to the time at which the probe is located closest to the sample because the time variation of $1/(1 + \sin \omega t)$ is maximum when $\Delta d/d_0 \approx 1$.

Supplementary References

[1] Suzuki, Y. Recent progress in MEMS electret generator for energy harvesting. *IEEJ Trans.* **6**, 101 (2011).

[2] Boland, J., Chao, Y.-H., Suzuki, Y., & Tai, Y. C. Micro electret power generator. *Proc. 16th IEEE Int. Conf. Miro Electro Mechanical System* 538 (2003).

[3] Hinchet, R., Ghaffarinejad, A., Lu, Y., Hasani, J. Y., Kim, S.-W., & Basset, P. Understanding and modeling of triboelectric-electret nanogenerator. *Nano Energy* **47**, 401 (2018).

[4] Chiu, Y., & Lee, Y.-C. Flat and robust out-of-plane vibrational electret energy harvester. *J. Micromech. Microeng.* **23**, 015012 (2012).

[5] Noguchi, Y., Miyazaki, Y., Tanaka, Y., Sato, N., Nakayama, Y., Schmidt, T. D., Brütting, W., & Ishii, H. Charge accumulation at organic semiconductor interfaces due to a permanent dipole moment and its orientational order in bilayer devices. *J. Appl. Phys.* **111**, 114508 (2012).
